# Chronic pruritus without rash in the setting of blastocystis and *Helicobacter pylori* coinfection: A case report

**DOI:** 10.1016/j.jdcr.2026.04.038

**Published:** 2026-04-29

**Authors:** Leah E. Thomas, Mina Botros, Sarahi Mera, Ashley Elsensohn

**Affiliations:** a Loma Linda University School of Medicine, Loma Linda, California; b University of California Riverside School of Medicine, Riverside, California; c Department of Dermatology, Loma Linda University Health, Loma Linda, California

**Keywords:** Blastocystis, eosinophilia, gastrointestinal infection, H. pylori, Pruritus, tropical disease

## Introduction

Chronic pruritus, defined as itching lasting over 6 weeks, significantly impairs quality of life and presents a diagnostic challenge due to its broad differential diagnosis, including dermatologic, systemic, neurologic, hematologic, and psychogenic causes. Current guidelines emphasize a structured evaluation to exclude underlying systemic disease.^1^^,^^2^

Infectious etiologies, particularly gastrointestinal pathogens, are less commonly considered in patients with chronic pruritus without primary skin findings. *Blastocystis* species and *Helicobacter pylori* have been associated with dermatologic conditions such as urticaria and nonspecific pruritus; however, their role in isolated chronic pruritus remains incompletely defined.^3^^,^^4^ We present a case of chronic pruritus without rash in a patient with *Blastocystis* and *H pylori* infection, highlighting the need for a comprehensive diagnostic approach.

## Case report

A 56-year-old Spanish-speaking woman with a history of hypertension presented with a 4-year history of generalized, predominantly nocturnal pruritus without associated primary rash. Symptoms began in 2018 and were persistent despite treatment with emollients, topical corticosteroids, and oral antihistamines. The pruritus was severe, disrupting sleep and causing excoriations, but no primary lesions were present.

Initial evaluation from her primary physician between 2021 and 2023 included repeated laboratory testing with complete blood count, comprehensive metabolic panel, thyroid-stimulating hormone, erythrocyte sedimentation rate, and urinalysis, which were within normal limits. The complete blood count consistently demonstrated normal white blood cell and platelet counts, and renal function remained normal with an estimated glomerular filtration rate >100 mL/min/1.73 m^2^.

Review of systems was negative for constitutional, pulmonary, hepatic, renal, and neurologic symptoms. In the absence of systemic findings or laboratory abnormalities suggestive of hematologic or malignant processes, further evaluation such as peripheral blood smear or chest imaging was not pursued.

In December 2023, dermatologic evaluation revealed mild peripheral eosinophilia (0.5 × 10^9^/L; reference 0.0–0.4 × 10^9^/L). A broad systemic workup was performed to evaluate for secondary causes of chronic pruritus, consistent with guideline recommendations.^1^^,^^2^ Further systemic workup was performed, including testing for HIV, hepatitis A/B/C, fecal occult blood testing and tuberculosis, all of which were negative.

The patient denied new medications, supplements, or environmental exposures, and there was no history suggestive of contact dermatitis; patch testing was not performed. The patient disclosed frequent travel to Mexico to visit family.

In late 2023, dermatologic evaluation confirmed chronic pruritus without rash. Stool ova and parasite examination in February 2024 identified *Blastocystis* species (subtype 3). The patient was treated with metronidazole 750 mg three times daily for 10 days. During treatment, pruritus persisted but subsequently improved modestly.

Despite partial improvement, the patient continued to experience significant pruritus. In mid-2024, she developed new epigastric pain, prompting further evaluation. Initial *H pylori* testing in February 2024 was negative; however, repeat stool antigen testing in October 2024 was positive. She was treated with quadruple therapy (amoxicillin, clarithromycin, metronidazole, and pantoprazole), resulting in resolution of gastrointestinal symptoms and further improvement in pruritus, though symptoms persisted at a lower intensity.

Due to ongoing refractory pruritus, methotrexate 10 mg weekly with folic acid supplementation was initiated in December 2024, resulting in near-complete resolution. By early 2025, pruritus was no longer a primary complaint. Subsequent testing confirmed eradication of *H pylori*.

## Discussion

This case illustrates the complexity of chronic pruritus without rash and suggests a multifactorial etiology involving infectious and immunologic components. While *Blastocystis* and *H pylori* infections temporally correlated with partial symptom improvement following treatment, complete resolution occurred only after initiation of methotrexate, indicating that these pathogens were unlikely to be the sole drivers of disease.

*Blastocystis* subtype 3 is among the most commonly identified subtypes in humans and has been associated with both asymptomatic colonization and symptomatic infection, although its pathogenic role remains debated.^3^ Prior reports have linked *Blastocystis* infection to urticaria and pruritus, potentially mediated through immune activation.^3^ Similarly, *H pylori* has been implicated in various dermatologic conditions, possibly through systemic immune modulation and chronic inflammation.^4^

Persistent pruritus despite eradication of infectious triggers in this case may be explained by sustained immune dysregulation and neural sensitization. Chronic pruritus is increasingly understood as a neuroimmune condition mediated by type 2 cytokines, including interleukin (IL)-4, IL-13, IL-31, and IL-33, which can directly activate pruriceptive neurons and promote neuroimmune crosstalk.^2^^,^^5^^,^^6^ These pathways may remain dysregulated even after resolution of the inciting infection, resulting in continued itch signaling.^2^^,^^6^ In this patient, early eosinophilia further supports activation of a Th2-skewed immune response. The response to methotrexate validates an immune-mediated component, as its immunomodulatory effects and suppression of Th2 response likely attenuated persistent cytokine signaling sustaining pruritus.^7^

**Fig 1:**
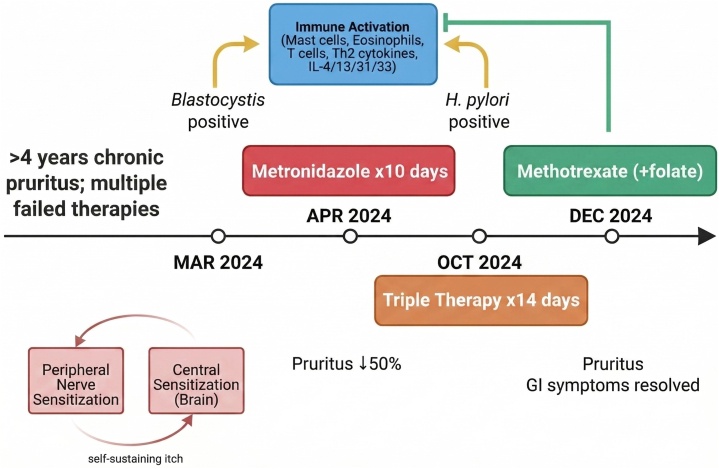
Clinical timeline of pruritus severity treatment and intervention with associated immune and neural pathways.

In addition, prolonged untreated pruritus may lead to peripheral and central sensitization of itch pathways, analogous to mechanisms described in chronic pain.^8^^,^^9^ Repeated stimulation over several years can induce neuroplastic changes, including heightened responsiveness of pruriceptive neurons and reduced inhibitory signaling, resulting in a self-sustaining itch state independent of the original trigger.^8^, ^9^, ^10^ This may explain why eradication of Blastocystis and H. pylori led only to partial improvement, whereas complete resolution required immunomodulatory therapy (Fig 1).

These findings highlight the importance of timely identification and treatment of potential underlying triggers. Delayed eradication may allow for the development of chronic neuroimmune dysregulation, in which removal of the inciting factor alone is insufficient for complete symptom resolution.

Case limitations included a resource-limited setting and language barriers; additionally, dermatology services were unavailable until 2023. Consequently, further diagnostics like peripheral blood smears or chest imaging were deferred without clear clinical indications.

## Conclusion

Chronic pruritus without rash requires a comprehensive and systematic evaluation. This case suggests that *Blastocystis* and *H pylori* infection may represent contributing factors in select patients, particularly when accompanied by eosinophilia or gastrointestinal symptoms. However, complete symptom resolution may require immunomodulatory therapy, highlighting the multifactorial nature of the disease. Consideration of infectious triggers alongside broader systemic and immunologic causes may improve diagnostic accuracy and management in refractory cases.

## Conflict of interest

Elsensohn attests to completing the Academy’s electronic disclosure form and serves in one or more of the following roles: Chair or member of an Academy Council, Committee, Task Force, Ad Hoc Task Force, or Work Group; contributor to CME/CCP activities (including planning committee member, session director, faculty, presenter, moderator, author, peer reviewer, or staff). The other coauthors have no conflict of interest to declare.

## References

[1] Butler D.C., Berger T., Elmariah S. (2024). Chronic pruritus: a review. JAMA.

[2] Weisshaar E., Szepietowski J.C., Dalgard F.J. (2019). European S2k guideline on chronic pruritus. Acta Derm Venereol.

[3] Bahrami F., Babaei E., Badirzadeh A., Riabi T.R., Abdoli A. (2020). Blastocystis, urticaria, and skin disorders: review of the current evidences. Eur J Clin Microbiol Infect Dis.

[4] Guarneri C., Ceccarelli M., Rinaldi L., Cacopardo B., Nunnari G., Guarneri F. (2020). Helicobacter pylori and skin disorders: a comprehensive review. Eur Rev Med Pharmacol Sci.

[5] Jha MK, Han Y, Liu Z (2025). Type 2 cytokines pleiotropically modulate sensory nerve architecture and neuroimmune interactions to mediate itch. J Allergy Clin Immunol.

[6] Du LX, Zhu JY, Mi WL (2022). Cytokines and chemokines modulation of itch. Neuroscience.

[7] Chan ES, Cronstein BN (2010). Methotrexate—how does it really work?. Nat Rev Rheumatol.

[8] Mahmoud O, Oladipo O, Mahmoud RH, Yosipovitch G (2023). Itch: from the skin to the brain—peripheral and central neural sensitization in chronic itch. Front Mol Neurosci.

[9] Misery L, Pierre O, Le Gall-Ianotto C (2023). Basic mechanisms of itch. J Allergy Clin Immunol.

[10] Andersen HH, Akiyama T, Nattkemper LA (2018). Alloknesis and hyperknesis-mechanisms, assessment methodology, and clinical implications of itch sensitization. Pain.

